# Isomorphous Substitution in ZSM-5 in Tandem Methanol/Zeolite Catalysts for the Hydrogenation of CO_2_ to Aromatics

**DOI:** 10.1021/acs.energyfuels.3c03755

**Published:** 2024-01-09

**Authors:** Dhrumil R. Shah, Iman Nezam, Wei Zhou, Laura Proaño, Christopher W. Jones

**Affiliations:** † School of Chemical & Biomolecular Engineering, Georgia Institute of Technology, 311 Ferst Dr., Atlanta, Georgia 30332, United States; ‡ State Key Laboratory of Physical Chemistry of Solid Surfaces, Collaborative Innovation Center of Chemistry for Energy Materials, National Engineering Laboratory for Green Chemical Productions of Alcohols, Ethers and Esters, College of Chemistry and Chemical Engineering, Xiamen University, Xiamen 361005, P. R. China

## Abstract

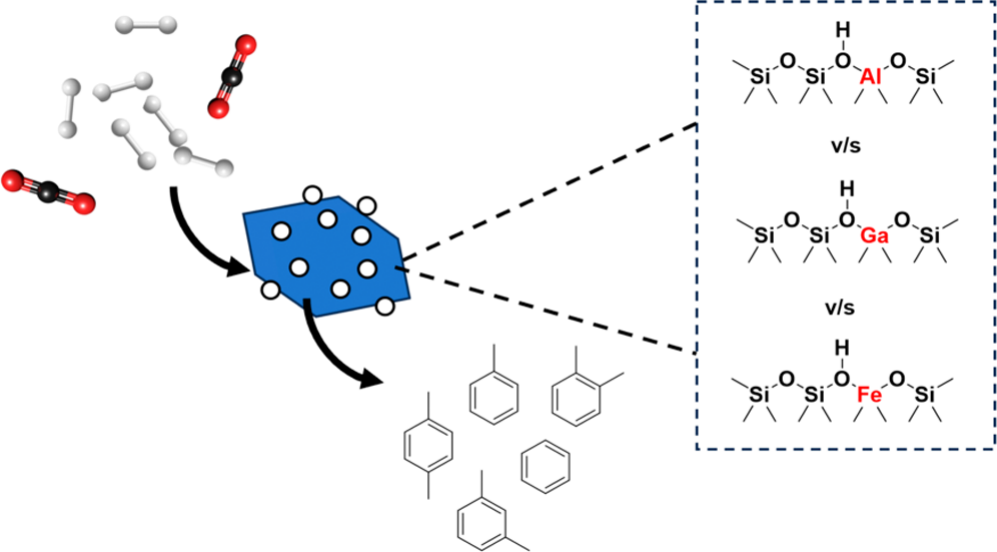

Intensified reactors for conversion of CO_2_ to methanol (via hydrogenation) using metal oxide catalysts coupled with methanol conversion to aromatics in the presence of zeolites (e.g., H-ZSM-5) in a single step are investigated. Brønsted acid sites (BAS) in H-ZSM-5 are important sites in methanol aromatization reactions, and correlations of the reactivity with zeolite acid properties can guide reaction optimization. A classical way of tuning the acidity of zeolites is via the effect of the isomorphous substitution of the heteroatom in the framework. In this work, H-[Al/Ga/Fe]-ZSM-5 zeolites are synthesized with Si/*T* ratios = 80, 300, affecting the acid site strength as well as distribution of Brønsted and Lewis acid sites. On catalytic testing of the H-[Al/Ga/Fe]-ZSM-5/ZnO-ZrO_2_ samples for tandem CO_2_ hydrogenation and methanol conversion, the presence of weaker Brønsted acid sites improves the aromatics selectivity (CO_2_ to aromatics selectivity ranging from 13 to 47%); however, this effect of acid strength was not observed at low *T* atom content. Catalytic testing of H-[B]-ZSM-5/ZnO-ZrO_2_ provides no conversion of CO_2_ to hydrocarbons, showing that there is a minimum acid site strength needed for measurable aromatization reactivity. The H-[Fe]-ZSM-5–80/ZnO-ZrO_2_ catalyst shows the best catalytic activity with a CO_2_ conversion of ∼10% with a CO_2_ to aromatics selectivity of ∼51%. The catalyst is shown to provide stable activity and selectivity over more than 250 h on stream.

## Introduction

Since the dawn of industrialization, global CO_2_ emissions have been on the rise, with current anthropogenic activities leading to an average addition of 4.9 GtC per year.^1^ CO_2_ must be removed from the environment, and simultaneously we must decrease the emissions of CO_2_ from industrial processes and human activities.^2^ While efforts are being made to make CO_2_ capture more economically feasible by improving technology,^3^ CO_2_ utilization could help make CO_2_ capture more economically attractive by delivering an intrinsically valuable product.^4^ Sun et al.^5^ studied plasma-catalytic CO_2_ hydrogenation using Pd/ZnO catalyst to get CO, which is an important intermediate in the Fischer–Tropsch synthesis of hydrocarbon fuels. Cyanation of benzylic C–N bonds is another example of CO_2_ utilization studied by Yan et al.^6^ Some of the largest petrochemical products that are essential to modern life are derived from aromatic chemicals. Currently, more than half of aromatics are sourced from catalytic reforming of crude oil, followed by steam cracking of naphtha.^7^ To this end, more eco-friendly processes for producing aromatics, whereby CO_2_ is the source of carbon, could be potentially beneficial.

CO_2_ hydrogenation has the potential to provide a range of products based on the choice of catalyst. Some examples are LPG,^8^ light olefins,^9,10^ aromatics,^11^ etc. Wang et al. suggest that CO_2_ to aromatics may be a more attractive pathway, as the overall change in Gibbs free energy in synthesis is lower than the synthesis of other products.^12^ With a versatile range of aromatic products,^7^ and the largest demand for monomers for polymer production, there is strong motivation for the synthesis of these molecules from a renewable or waste source, unlike the current synthesis method that depends on virgin fossil feedstocks. To this end, CO_2_ hydrogenation to produce aromatics could prove to be an important synthesis pathway to meet the increasing demand for aromatics as well as utilizing industrially emitted CO_2_.

CO_2_ conversion to aromatics can be achieved in multiple separate steps, for example, CO_2_ hydrogenation toward methanol or alkanes/alkenes, followed by zeolite-catalyzed conversion in a second reactor to aromatics. Recently, researchers have sought to develop an intensified process completing both steps in a single reactor.^13^ In one manifestation of an intensified process, CO_2_ hydrogenation to produce aromatics can be achieved by utilizing tandem catalysts involving the conversion of CO_2_ to methanol (MeOH) over a metal–metal oxide catalyst followed by aromatization over an H-ZSM-5 catalyst.^13^ An example of this tandem catalyst is ZnO-ZrO_2_ paired with H-ZSM-5. While the conversion of CO_2_ to MeOH is an exothermic reaction, the aromatization reaction is endothermic. However, pairing the reactions in a single-bed reactor is not trivial, as a temperature regime (∼300–340 °C) must be used that is not ideal for either individual reaction. This range is higher than the ideal temperature regime for methanol synthesis, as CO production is favored at these higher temperatures, and lower than the ideal temperatures for the methanol aromatization reaction. This typically results in side reactions, leading to the formation of significant amounts of CO and paraffins and limiting product yield. Hence, there is a motivation to improve the selectivity of CO_2_ to aromatics by changing the properties of the tandem catalysts, while learning about the structure/performance attributes of the zeolite components of the tandem reaction.

**Figure 1:**
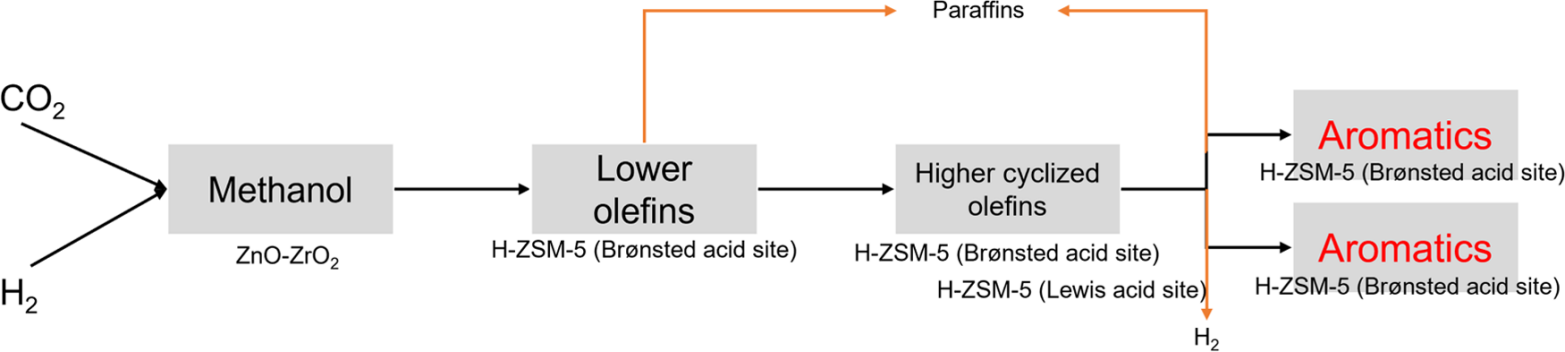
Pathway summarizing different types of reactions involved in the conversion of methanol or C2–C4 hydrocarbons to aromatics in H-ZSM-5.

H-ZSM-5 is highly selective toward the synthesis of light aromatics from methanol. A simplified CO_2_ to hydrocarbon reaction pathway is summarized in Figure 1.^13−15^ Ilias et al. and Biscardi et al. in their recent studies showed that methanol converts to lower olefins and further oligomerizes to higher cyclized olefins. These higher olefins convert to aromatics by the removal of hydrogen by a H-transfer pathway or dehydrogenation. This affects the yield of aromatics as well as the production of paraffins as side products. While the H-transfer reaction occurring over Brønsted acid sites occurs via the transfer of hydrogen atoms from higher olefins to lower olefins producing aromatics as well as paraffins, dehydrogenation leads to the removal of hydrogen atoms as H_2_ and has been proposed to occur because of the presence of some particular Lewis acid sites coupled with Brønsted acid sites.^13,16^ For high selectivity, it has thus been suggested that the right balance of these Lewis and Brønsted acid sites is important in promoting the highest rates of dehydrogenation relative to the rates of H-transfer.

To alleviate these side reactions, multiple modifications of H-ZSM-5 have been explored. In unmodified ZSM-5, the aromatics are alkylated to heavier aromatics upon diffusion to external sites and blocking the external acid sites helped in enhancing the BTX selectivity. A capsule-type Zn-doped ZSM-5 catalyst was designed to render the external acid sites less active (sites responsible for methylation of aromatics), with the Zn-doping promoting dehydrogenation by introducing Lewis acid sites and improving conversion rates.^17^ This led to aromatic selectivity of nearly 70% of hydrocarbons synthesized at conversions of more than 20%. Zhang et al.^18^ observed that doping with Zn and P improved the aromatics selectivity from undoped H-ZSM-5. In this kind of doping, it was observed that the density of medium-strong acid sites increased, which the authors suggested promoted dehydrogenation and enhanced the conversion of LPG to aromatics. Wang et al.^19^ showed that by blocking the external acid sites of H-ZSM-5, the selectivity to the valuable BTX aromatics significantly increased for CO_2_ hydrogenation, as the modification prevents further methylation of the aromatics as they diffused out of the zeolite crystal.

Various isomorphous substituted ZSM-5 catalysts have been synthesized where the Al in the framework has been replaced with similar-sized tetrahedral atoms such as B, Ga, and Fe.^20,21^ Replacement of Al with other heteroatoms has long been used as a method to change the acid strength and distribution of Lewis and Brønsted acid sites in zeolites in a range of reactions, including methanol conversion reactions.^22,23^ The isomorphous substitution of ZSM-5 is known to significantly affect the properties of ZSM-5 and this has been used as a catalyst design tool for many decades.^20,21,24,25^ Over the years, numerous studies have suggested that the Brønsted acid site strengths have the following order: H-[Al]ZSM-5 > H-[Ga]ZSM-5 > H-[Fe]ZSM-5 ≫ H-[B]-ZSM-5.^26^ Multiple reactions like hexane cracking and propene oligomerization,^21^ dehydration of ethanol,^25^ production of ethylene and propylene from methanol,^27^ and others have been investigated over different framework substituted zeolites. However, the effect of the isomorphous substitution of ZSM-5 as part of tandem catalysts for the conversion of CO_2_ to aromatics has not yet been explored in the literature. The isomorphous substitution modifies the nature of acid sites as well as affects the in-framework and out-of-framework *T* atom distributions, affecting the reactivity and selectivity to BTX products, as these *T* atoms lead to the formation of acid sites where the aromatization, alkylation and other key reactions occur. Hence, in this work, we explored the impact of the isomorphous substitution of ZSM-5 with Al, Ga, Fe, and B in tandem with a known ZnO-ZrO_2_ methanol synthesis catalyst on the hydrogenation of CO_2_ to aromatics.

## Experimental Methods

### Synthesis of Materials

#### Synthesis of H-[Al]-ZSM-5

The H-[Al]-ZSM-5–80 and H-[Al]-ZSM-5–300 were synthesized using the hydrothermal method.^28^ The reactants were mixed in the following molar ratios of each component: 1.0 SiO_2_/0.45 TPAOH/*x* NaAlO_2_/50 H_2_O, with the value of *x* = 0.0125 and 0.0033 based on Si/Al = 80 and 300, respectively. Tetrapropylammonium hydroxide (TPAOH) (25% in water, Thermo Scientific Chemicals) is mixed with deionized (DI) water and stirred for at least 10 min. To this stirring mixture was added tetraethylorthosilicate (TEOS) (≥99%, Sigma-Aldrich) and was then hydrolyzed by the addition of NaAlO_2_ (technical grade, Sigma-Aldrich). This mixture was then stirred vigorously for more than 3 h, followed by transfer to a Parr autoclave and heating in an oven preheated at 170 °C for 2 days. This crystallized zeolite was then washed 3 times with water (mixed with ∼30 mL DI water, followed by running the sample in a centrifuge at 8000 rpm × 10 min) and dried overnight at 80 °C and later calcined at 550 °C (heated at a ramping rate of 2 °C/min) for 6 h. The Na-form zeolite (Na-[Al]-ZSM-5) was then converted to its protic form (H-[Al]-ZSM-5) by ion exchanging the ZSM-5 with 100 mL of 1.0 M NH_4_NO_3_ (≥98%, Sigma-Aldrich) three times at 80 °C for 4 h each time followed by drying at 80 °C overnight and calcining the zeolite.

#### Synthesis of H-[B/Ga/Fe]ZSM-5

The H-[*T*]-ZSM-5-*x* (*T* = B/Ga/Fe; *x* = 80, 300) was synthesized by the method reported by Kim et al.^29^ The initial reactants were mixed in the ratio: 1.0 SiO_2_/0.32 TPAOH/*x* T(X_3_)_3_/45.4 H_2_O, where *x* = 0.0125 and *x* = 0.0033 and T(X)_3_ refers to the nitrate salts of B/Ga/Fe {B(OH_3_)_3_/Ga(NO_3_)_3_/Fe(NO_3_)_3_} (≥99.95% trace metals basis, Sigma-Aldrich) based on the zeolite being synthesized. TPAOH and DI water were rigorously mixed, followed by the addition of the silica source, TEOS. T(NO_3_)_3_ was added immediately along with TEOS. The mixture was stirred for about 24 h and then was transferred into a Teflon-lined Parr autoclave and kept for crystallization in an oven preheated to 150 °C for 4 days. The solid products were then centrifuged, washed with water (mixed with ∼30 mL DI water, followed by running the sample in a centrifuge at 8000 rpm × 10 min) at least 3 times, and then dried at 80 °C overnight. This sample was then calcined at 550 °C (heated at a ramping rate of 2 °C/min) for 6 h.

#### Synthesis of ZnO-ZrO_2_

ZnO-ZrO_2_ was synthesized by the coprecipitation method reported by Wang et al.^30^ Zn/Zr ratio of 1:6 was chosen based on previous studies done on the metal oxide catalyst.^11,31^ First, 0.6 g of Zn(NO_3_)_2_·*x*H_2_O (≥98%, Sigma-Aldrich) and 5.43 g of ZrO(NO_3_)_4_·6H_2_O (≥98%, Sigma-Aldrich) were dissolved in 100 mL of deionized water and stirred at 70 °C. To this stirring mixture, 3.06 g of (NH_4_)_2_CO_3_ (ACS grade, Sigma-Aldrich) dissolved in 100 mL of deionized water was added drop by drop. The mixture was stirred for 2 h, and then it was cooled back to room temperature. The solid product was recovered by vacuum filtration, washed with DI water, and dried overnight at 80 °C. This catalyst was then calcined in air at 500 °C for 5 h.

### Catalyst Characterization

#### Ammonia Temperature-Programmed Desorption (NH_3_-TPD)

NH_3_-TPD measurements were performed to estimate the total acid site density for H-ZSM-5. These experiments were conducted in Micromeritics Autochem II automated chemisorption analyzer. A known amount of sample (∼50 mg) was pretreated at 400 °C (ramping at 10 °C/min) in helium atmosphere for 1 h. Later, the zeolites were exposed to NH_3_ for sorption at 40 °C until the TCD sensor stabilized. Temperature-programmed desorption was performed at a rate of 10 °C/min until 800 °C. The desorption of ammonia was tracked using the built-in TCD sensor, and the overall acid sites were estimated assuming each acid site would desorb not more than one ammonia molecule.

#### Isopropylamine Temperature-Programmed Desorption (IPA-TPD)

IPA-TPD experiments were performed over an in-house fixed bed setup connected to a mass spectrometer (Pfeifer Vacuum GSD-320) to measure real-time concentrations. A known mass of ZSM-5 (∼200–300 mg) was pelletized and loaded onto the fixed bed. The ZSM-5 was next pretreated with flowing N_2_ at 400 °C for 1 h (ramping at 10 °C/min). The sample was then cooled to 100 °C. Isopropylamine was then added with 3 × 50 μL injections, and its vapors were carried over the catalyst using N_2_ as the carrying medium. Once sufficient time was given for the removal of the nonadsorbed IPA (as confirmed by the absence of IPA signals on the mass-spectrometer), the sample was heated at a constant rate of 5 °C/min up to 700 °C using a temperature program under flowing N_2_. The signals corresponding to propylene (*m*/*e* = 41), isopropylamine (*m*/*e* = 42), and ammonia (*m*/*e* = 17) were tracked in real time with the Faraday sensor on the online mass spectrometer connected to the outlet of the fixed bed. Using the ionic displacement vs time curve, a concentration vs time curve was made for propylene and the overall moles, and hence, the concentration of Brønsted acid sites on the ZSM-5 was calculated by integrating this curve. Brønsted sites produce propylene and ammonia, whereas Lewis acid sites desorb isopropylamine.^32^

#### Composition, Structure, and Porosity Analysis

Nitrogen physisorption was conducted at 77 K on a BELSORP-max (MicrotracBEL). H-ZSM-5 samples were degassed under a vacuum at 250 °C for 12 h. Scanning electron microscopy (SEM, Hitachi SU8230) was performed on the catalyst sample for the measurement of catalyst particle size. XRD experiments were performed using Cu Kα radiation in a PANalytical XPert PRO α-1 diffractometer. Diffraction in the 2θ range of 5–90° was measured with a step size of ∼0.05°. To estimate the Si/(Al, Ga, Fe) ratio, ICP-OES analysis was performed by Galbraith Laboratories, Inc. (Knoxville, Tennessee). Scanning transmission electron microscopy (STEM) along with energy-dispersive X-ray spectroscopy were performed using a Hitachi HD2700. Samples were prepared using carbon-coated copper grids using an ethanol suspension. Temperature-programmed reduction of H_2_ (H_2_-TPR) was conducted using Micromeritics Autochem II automated chemisorption analyzer instrument. The samples were tested for H_2_ reduction until 800 °C.

#### Heteroatom Analysis

^71^Ga solid-state NMR (ssNMR) was measured for H-[Ga]-ZSM-5 using a Bruker Avance III 400 MHz spectrometer with a dwell time of 1 μs and a pulse delay of 0.5 s. Each sample was rotated at 12 kHz using a Bruker 4 mm MAS rotor, taking ∼32 000 scans.

To determine the nature of the Al content in H-[Al]-ZSM-5, ^27^Al ssNMR was measured using a Bruker Avance III 400 MHz spectrometer with a dwell time of 1 μs and a pulse delay of 1 s. The zeolite samples were packed in a 4 mm zirconia rotor and rotated at 12 kHz, and each run involved taking ∼8000 scans.

To probe the Fe content of H-[Fe]-ZSM-5, UV–vis diffuse reflectance spectroscopy (DRS) was performed by using a Cary 5000 UV/vis NIR spectrometer.

All of the zeolites were also studied for their Si distribution using ^29^Si ssNMR. This was performed using a Bruker AVIII-HD 300 MHz solid-state spectrometer. The zeolite samples were packed in a 4 mm zirconia rotor and rotated at 10 kHz. The dwell time was set at 16 μs with a pulse delay of 2 s. Each ssNMR run was measured for ∼4000 scans.

### Catalytic Testing

The tandem catalysts were tested using a fixed bed setup connected to an online GC, as shown in the Supporting Information (Figure S1). ZnO-ZrO_2_ and ZSM-5 powders were mixed in a 1:2 w/w ratio using a mortar and pestle for at least 15 min to aid uniform mixing. This mixed powder was then pelletized and sieved using sieves of mesh sizes 35 and 100. Then ∼200 mg of the pellets between these sizes were packed between beds of inert silicon carbide beads (Sigma-Aldrich, 200 mesh size) in a quarter-inch SS316 reactor tube. A heating jacket made from 12″ house-made aluminum blocks and two 550 W Chromalox cartridge heaters was used to heat the reactor tube to the required temperatures. A premixed gas (33% H_2_, 11% CO_2_, balance N_2_) provided by Matheson was used as feed to the reactor. This premixed gas was first transferred to a low-pressure tank until the pressure reached about 300 psi. Using a gas booster (Maximator DL-30-1), the gas was then transferred and stored at about 2000 psi in a high-pressure tank ready to be fed to the reactor. All of the flow rates were controlled using mass flow controllers (Brooks Instrument, SLA5850). The catalyst in the packed bed was pretreated at 400 °C for 3 h (ramping at 5 °C/min) using 60 mL/min with 5% H_2_ balanced by N_2_. After the pretreatment, the reactor was cooled down to 320 °C, followed by feeding the premixed gas stored in the high-pressure tank. The pressure within the reactor was regulated by means of a back-pressure regulator (Tescom ER3000). The temperature, pressure, and flow rates were controlled using a homemade LabVIEW program. The flow rate for each run was determined using the following formula
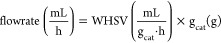


The product stream was analyzed using an online 7890 Agilent gas chromatograph (GC) that uses three columns (MolSieve, PoraBOND U, and CP-Wax) and two TCDs and one FID for quantifying various components present. The integrated GC curves were used to quantify the molar composition, and the following formulas were used for relevant calculations:
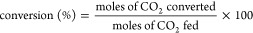

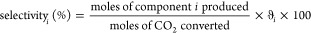

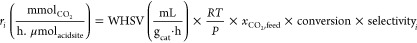
where ϑ_i_ is the stoichiometric coefficient for converting CO_2_ to component *i* and *x*_CO_2_, feed_ is the molar fraction of CO_2_ in the feed stream. The carbon balance was confirmed using the following equation:
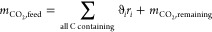
where *m*_CO_2_, feed_ is the molar flow rate of feed CO_2_ and *m*_CO_2_, remaining_ is the total molar flow rate of CO_2_ in the product stream.

## Results and Discussion

### Catalyst Characterization

**Figure 2:**
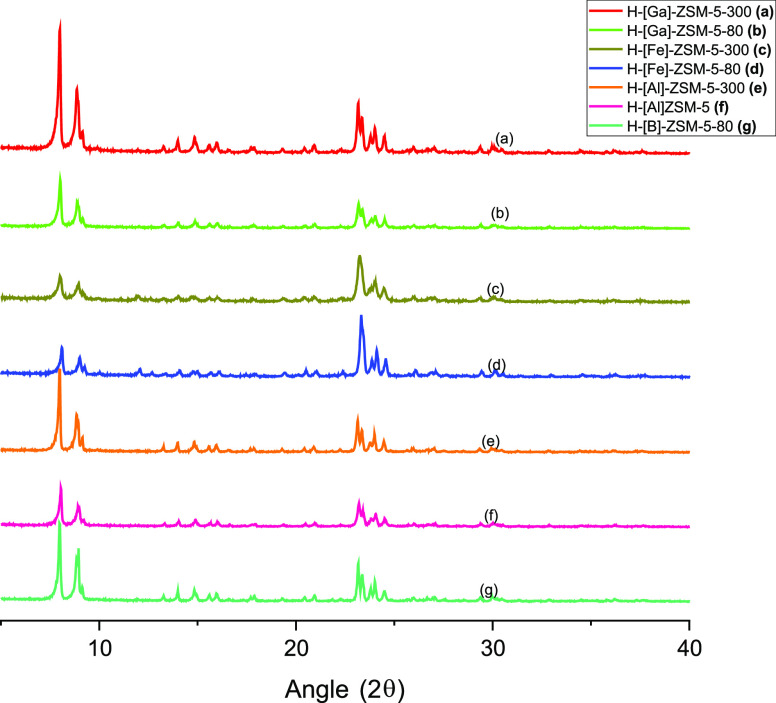
X-ray diffraction patterns of synthesized H-[*T*]-ZSM-5 materials.

Figure S2 shows the XRD patterns of the ZnO-ZrO_2_ methanol synthesis catalyst. The XRD pattern shows a tetragonal ZrO_2_ pattern, and no peaks relevant to ZnO crystals were observed. As STEM-EDS demonstrates the presence of Zn^2+^ ions (Figure 5), it appears that the Zn species are highly dispersed or in amorphous domains. The XRD pattern is consistent with characteristics reported in the literature for this mixed oxide synthesized in this way.^30,31,33^ There is no evidence of an amorphous phase in XRD or microscopy analysis (Figure S3). Similarly, the XRD patterns for all H-ZSM-5 catalysts used in this study are consistent with the MFI crystal structure and previous literature (Figure 2).^21,29,34,35^ The isomorphous substitution of H-ZSM-5 does not yield a significant change in the crystalline structure, as expected, since all of the concentrations of substituted *T* atoms are low and they all have similar valences. The H-[Fe]-ZSM-5 materials show a convoluted peak at ∼23° which is deconvoluted in Figure S10. These deconvoluted peaks match the location of the double peaks observed for other synthesized zeolites. The N_2_ physisorption experiments further affirm this, as the micropore volume does not change significantly with isomorphous substitution (Table 1). At the low heteroatom loadings used here (Si/T ∼ 80, 300), variations in the size of each *T* atom do not significantly impact porosity or crystallinity.

**Table 1:** Composition of H-ZSM-5 Samples and Measured Acidity Characteristics

ZSM-5	Si/*T* (synthesis)	Si/*T* (ICP-OES)	particle size (nm)	micropore volume (cm^3^/g)	NH_3_-TPD (μmol/g)	IPA-TPD (μmol/g)
H-[Fe]-ZSM-5–80	80	65	305	0.193	113	28
H-[Fe]-ZSM-5–300	300	241	222	0.186	24	∼0
H-[Ga]-ZSM-5–80	80	113	265	0.182	103	19
H-[Ga]-ZSM-5–300	300	243	248	0.185	20	12
H-[Al]-ZSM-5–80	80	103	272	0.182	106	44
H-[Al]-ZSM-5–300	300	262	329	0.184	24	24
H-[B]-ZSM-5–80	80	64	235	0.165	117	37

For series reactions,^36^ especially those involving transport between different reaction zones (e.g., two pore systems in a zeolite, or in this case, two distinct catalyst phases, ZnO-ZrO_2_ and H-ZSM-5), the crystal size and shape can significantly influence the reactivity. To this end, scanning electron microscopy (SEM) was used to assess the crystal size of the ZSM-5 samples. Figure S3 shows the SEM images of the zeolites with the distribution of crystal sizes and shapes. The average crystal size does not vary significantly across the zeolites, as shown in Table 1. The surface morphologies, as shown in the SEM images, do not differ significantly and are not expected to affect the reaction rates by themselves. However, it was interesting to see that the surface morphology looks to be subtly smoother for the higher Si/*T* ratios than for the lower Si/*T* ratios. His difference in surface morphology does not affect the overall micropore volume (Figure S4), which is consistent across all of the zeolites. This result paired with the XRD data led us to believe that the slightly varying surface morphology does not significantly affect the catalytic results.

Figure S5 shows the results of ^27^Al ssNMR of synthesized H-[Al]-ZSM-5. A single prominent peak at ∼54 ppm was observed, identified as the tetrahedral Al atom present in the framework of H-[Al]-ZSM-5. This implies that the synthesized zeolites have very small quantities of extraframework Al atoms.^37^^71^Ga ssNMR results for H-[Ga]-ZSM-5 shown in Figure S6 imply that almost all of the Ga atoms have been incorporated into the framework, as expected from the synthesis. The single peak at ∼150 ppm affirms so.^38,39^ Fe, being paramagnetic in nature, cannot be analyzed by NMR. Hence, the Fe distribution in the zeolite framework was studied by using UV/vis DRS (Figure S7). Both H-[Fe]-ZSM-5–80 and H-[Fe]-ZSM-5–300 show peaks at ∼211 and ∼252 nm, which point to the presence of primarily isolated Fe atoms, with H-[Fe]-ZSM-5–80 showing larger peaks due to higher Fe content. UV/vis for H-[Fe]-ZSM-5–80 also has a convoluted peak at ∼300 ppm, which indicates a small presence of iron oxides.^40,41^

All of the synthesized zeolites also show a similar Si distribution, as confirmed by ^29^Si ssNMR (Figure S8). The peak at −114 ppm is attributed to the Si(0T) group while the small peak at −104 ppm is due to the presence of silanols.^42^ Upon performing deconvolution of the peaks, a small peak is observed at ∼ −108 ppm, which is attributed to the Si(1T) group, which is present due to the small concentration of *T* atoms in the zeolite framework. From these heteroatom studies, we hence confirm that all of the zeolites synthesized for this study have similar structures, and hence, the Al/Ga/Fe/B atoms have been successfully incorporated into the framework.

**Figure 3:**
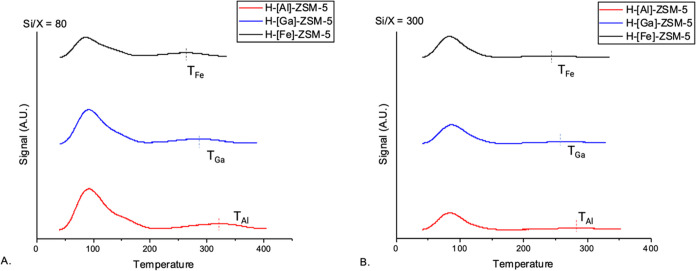
NH_3_-TPD profiles of synthesized H-[*T*]-ZSM-5 (*T* = Al/Ga/Fe). (A) Si/*T* = 80, (B) Si/*T* = 300.

The temperature-programmed desorption of ammonia is one of the most commonly used methods for the estimation of acid site densities in zeolites.^43^Figure 3 shows the NH_3_-TPD traces for the various synthesized ZSM-5s. All of the NH_3_-TPD traces show two distinct peaks: one being the weak acid sites that desorb at lower temperatures (∼100 °C), while the other represents strong acid sites, desorbing NH_3_ at higher temperatures (265–320 °C).^20^ The area of the separate peaks was calculated after the deconvolution of the peaks. Figure 3A shows the NH_3_-TPD results for the Si/*T* ratio of 80. MFI zeolites at low Si/*T* ratios show higher acid site densities, as expected, due to more *T* atoms per unit cell. The strong acid sites show that the temperature for desorption of ammonia *T*_des_ follows the order: *T*_des (*T*=Al)_ > *T*_des (*T*=Ga)_ > *T*_des (*T*=Fe)_, affirming the expected relative acid site strengths of each of the ZSM-5s. This assertion can be made given the similar crystal sizes and acid site densities across the various materials. It is to be noted that the strong acid site densities do not differ significantly across the 3 isomorphous substituted MFIs (the difference being less than 5%), at an average acid site density of ∼107 μmol/g. In the case of high Si/*T* ratios of 300 (Figure 3B), acid site densities are very low, as expected. All of those catalysts have acid site densities of ∼20 μmol/g, with the difference in acid site densities being less than instrument error.

The Brønsted acid sites present in H-ZSM-5 are key sites for aromatization reactions. To probe the distribution of Lewis vs Brønsted acid sites of the zeolites, IPA-TPD experiments were used to complement the total acidity measured by NH_3_-TPD. Prior work has shown that isopropylamine desorbed from a Brønsted acid site is dissociated to give propylene and ammonia, which can be detected by mass spectrometry.^44^Table 1 shows the key characteristics of the H-ZSM-5 samples, including the target and measured Si/*T* ratios, total acidity as measured by NH_3_ TPD, and Brønsted acidity assessed by IPA-TPD. For the Si/*T* = 300 samples, the total acidity is the same, with the Al-MFI having exclusively Brønsted acid sites. The Ga-substituted material shows an even mixture of Brønsted and Lewis sites. Gallium-based zeolites often produce a mixture of Lewis/Brønsted acid sites, owing to the propensity for Ga to leave the zeolite framework or form metal oxide clusters during synthesis or pretreatment.^45^ Brønsted acid sites seem to be very low (below detection levels) in the case of H-[Fe]-ZSM-5–300. However, there is the presence of significant Brønsted acid sites in the case of H-[Fe]-ZSM-5–80. The acid site distribution appears very similar for all of the Si/*T* = 80 zeolites, with the low Brønsted acid site density for H-[Ga]-ZSM-5–80 rationalized as explained above.

Figure S9 shows the H_2_-TPR of the zeolites synthesized. None of the zeolites apart from H-[Fe]-ZSM-5–80 showed any signs of reduction. The temperature of reduction at ∼350 °C signifies reduction of some Fe_2_O_3_ potentially present in H-[Fe]-ZSM-5–80 to lower oxidation states.^46,47^ This temperature is lower than the pretreatment temperature for catalytic testing, which leads to the possibility of forming Fe_3_O_4_ or FeO oxide phases during the reaction.

The overall characteristics of the ZSM-5 samples are summarized in Table 1. Prior work on methanol conversion over zeolite catalysts has shown that the zeolite topology, crystal size, acid site density, and nature of the acid sites all can influence the reactivity.^13,48^ In the experimental design here, we sought to hold constant the zeolite topology, crystal size, and total acid site density, while changing the strength and potentially distribution of the acid sites.^45,49^ With these zeolites in hand, composite catalysts composed of each zeolite individually mixed with a ZnO-ZrO_2_ methanol synthesis catalyst were deployed in the tandem hydrogenation of CO_2_ to aromatics.

### Catalytic Testing

Figure S12 shows the catalytic performance of H-[*T*]-ZSM-5 for the CO_2_ hydrogenation. As expected, the synthesized zeolites cannot convert CO_2_ effectively to methanol on their own. No significant conversion to products was observed in an empty tube or a SiC-filled reactor tube at steady state. CO_2_ conversions are also negligible across all of the zeolites, with any conversion primarily leading to CO production, with a small amount of methanol or methane also produced. This suggests the presence of minor amorphous metal oxides formed, perhaps during the calcination of the zeolites.^50^Figure S11 shows the catalytic performance of ZnO-ZrO_2_ alone, without zeolite. Methanol and CO are the primary products from the CO_2_ hydrogenation over the metal oxide catalyst. Considering the rich literature on the conversion of methanol to aromatics, and the inability for direct conversion of CO_2_ over zeolites, the production of methanol over ZnO-ZrO_2_ further affirms that aromatics are synthesized over ZnO-ZrO_2_/H-[*T*]-ZSM-5 via a methanol-mediated pathway, i.e., CO_2_ is converted to aromatics with methanol being one of the key intermediates in the conversion.^13,30,31^

**Figure 4:**
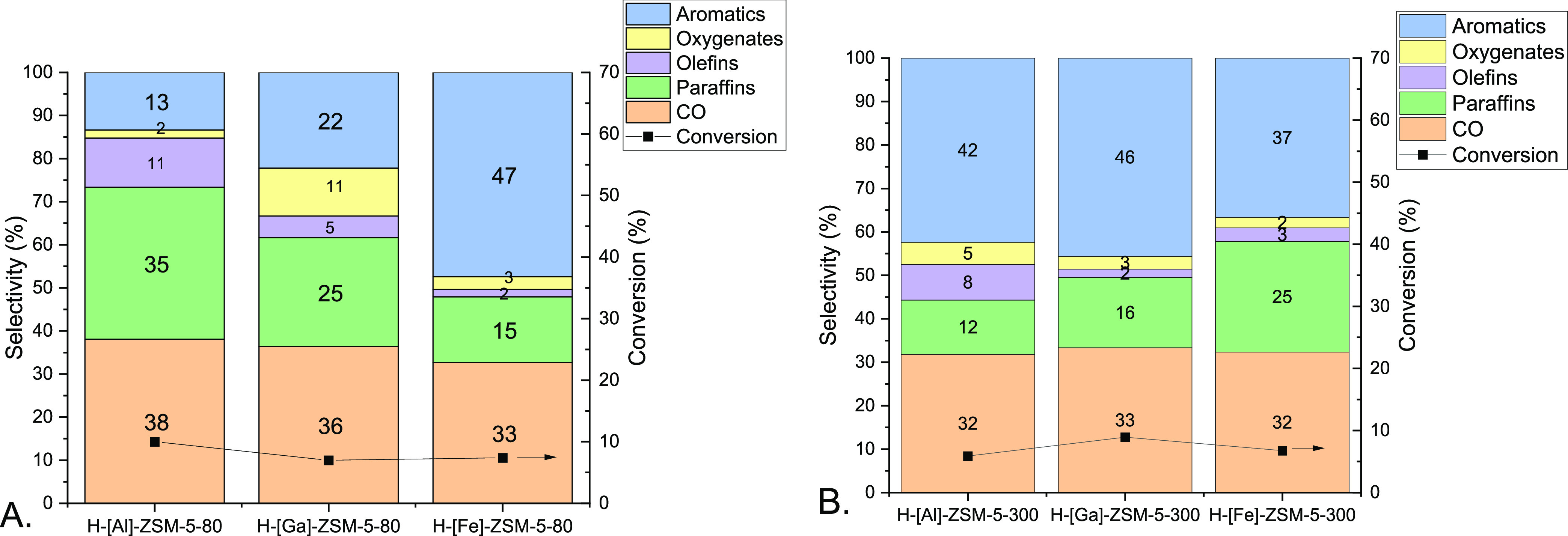
Product selectivities during CO_2_ conversion over H-ZSM-5 in tandem with ZnO-ZrO2. (A) Si/*T* = 80, (B) Si/*T* = 300. *T* = 320 °C, *P* = 600 psi, CO_2_/H_2_/N_2_ = 11:33:56, WHSV = 7200 mL g_cat_^–1^ h^–1^.

Figure 4 shows the catalytic performance of the ZnO-ZrO_2_/H-[*T*]-ZSM-5-(80,300) catalysts for CO_2_ hydrogenation. In each case, the WHSV has been modified such that the conversion remains in a similar range and all CO_2_ conversions are below 10%, seeking to approximate a differential reactor. The bar chart in Figure 4 (error of estimation is recorded in Table S2) allows for some immediate observations to be made. First, all catalysts produce approximately the same amount of CO, suggesting that the reverse water gas shift (RWGS) reaction proceeds to a similar extent over all catalysts. As this is influenced primarily by the methanol synthesis catalyst and reaction conditions, it is perhaps not surprising. There is a subtle enhancement of the RWGS reaction observed at a lower Si/*T* ratio (tandem catalyst ZnO-ZrO_2_/H-[*T*]-ZSM-5, Si/*T* = 80), i.e., higher acid site density (Figure 4A).^33^ A second observation is that the selectivity for olefins (C_2_–C_7_) and oxygenates does not vary substantially across the catalysts, with two exceptions that will be discussed later. In contrast, a clear trade-off between aromatics and paraffins can be observed across all catalysts, and this is influenced both by acid site density (Si/*T*) and the nature of the acidity (Al, Ga, Fe). This represents the balance between H-transfer reactions, producing alkanes, and dehydrogenation reactions, producing H_2_, in the pathways to aromatic products. Prior work by others has shown an impact of zeolite acid site density in these tandem reactions,^33^ and our prior work identified an optimal Si/Al range for tandem reactions with ZnO-ZrO_2_/H-[Al]-ZSM-5.^48^ Here, at Si/*T* = 80, the Fe substituted zeolite gave the highest selectivity to aromatics while minimizing the paraffin production. From there, paraffin selectivity seems to correlate with zeolite acid strength, with stronger acids producing more paraffins and less aromatics. However, one cannot simply draw this conclusion without considering the role of the acid site type. At this Si/*T* ratio, the Fe, Ga, and Al catalysts have mostly Lewis acid sites (81%, Ga and 75%, Fe) and balanced Brønsted/Lewis site distributions (40:60 B/L, Al). While H-[Fe]-ZSM-5–80 does have potential iron oxide species, as seen in our H_2_-TPR results, there is little evidence of its beneficial effects to aromatization reaction, as pointed out in another study done by Brabec et al.,^51^ nor any evidence of new side products. The influence of any iron oxide phase and potential iron species reduction will be further investigated in a future study. From these results, one can infer that tandem catalysts with zeolite domains containing weak Brønsted acid site distributions can give good aromatic selectivity, which is a trend not previously noted in the literature.

**Figure 5:**
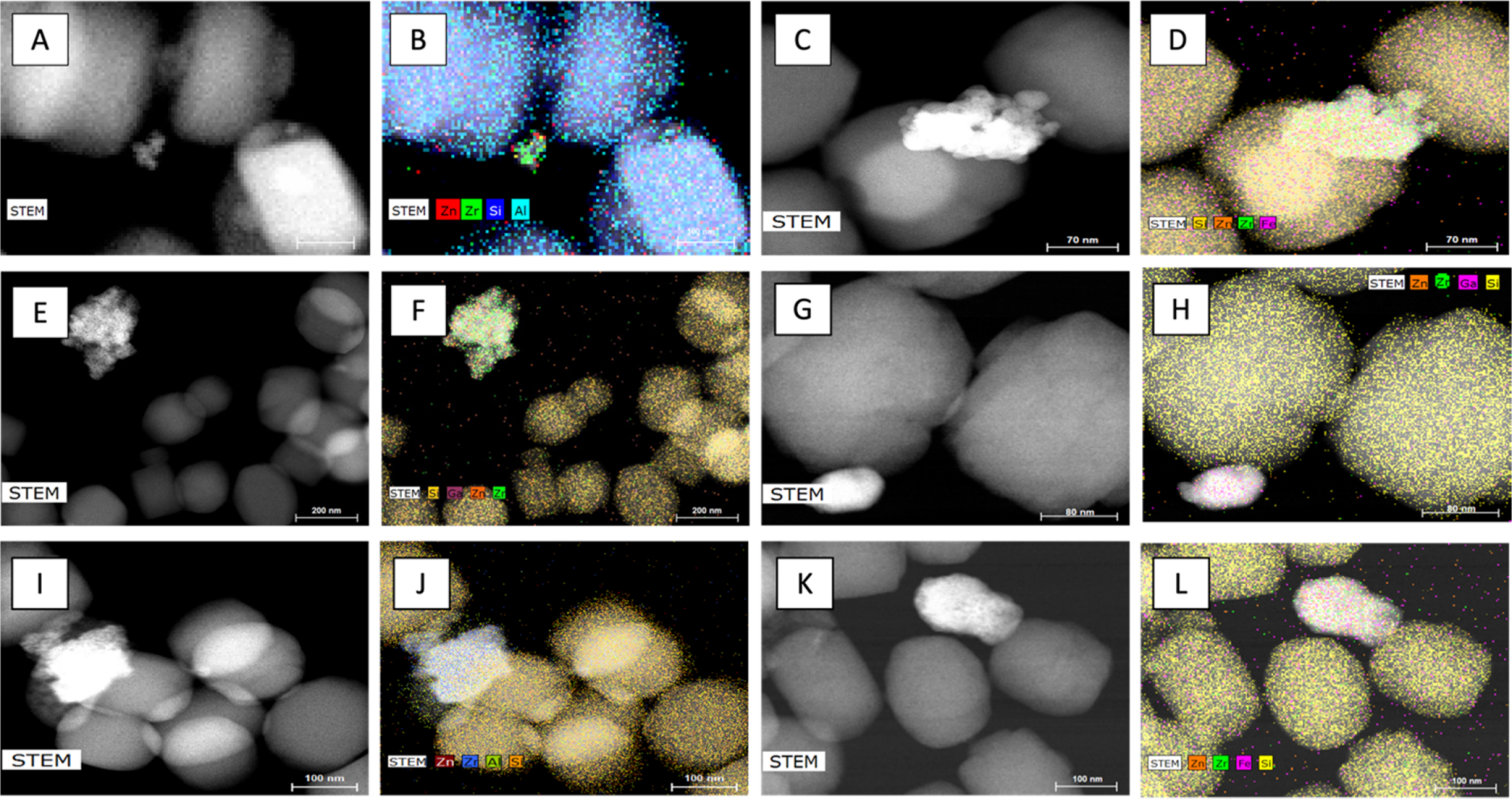
STEM-EDS for ZnO-ZrO_2_/H-[*T*]-ZSM-5 postcatalytic testing. (A, B) *T* = Al, Si/*T* = 300, (C, D) *T* = Fe, Si/*T* = 300, (E, F) *T* = Ga, Si/*T* = 300, (G, H) *T* = Ga, Si/*T* = 80, (I, J) *T* = Al, Si/*T* = 80, (K, L) *T* = Fe, Si/*T* = Fe.

To probe whether there were effects of significant ion migration from the metal oxide into the zeolite pores and framework, HAADF-STEM studies were performed. In these experiments, the Zn, Zr, and T atoms were tracked, and the results are shown in Figure 5. It appears that no significant ion migration occurred within the time frame of the catalytic study (the catalyst was run for 24 h or less), though migration over longer time scales may still occur. This suggests that no additional acid sites were developed due to ion migration during the reaction duration and the aromatization reaction thus depends primarily on the Lewis and Brønsted acid sites that have been incorporated in the zeolites by design in this study.

There were two noteworthy observations regarding the olefin or oxygenate selectivities discussed above. First, we see that the H-[Al]-ZSM-5 catalyst gave significantly more olefins than did the other two catalysts. This could be attributed to higher β-scission reaction rates, as observed for stronger acid sites by Mehla et al.^52^ There is also potential for enhanced H-transfer due to higher acid site strength.^53^ Second, it is notable that Ga-MFI produces more oxygenates than the other catalysts. Based on the mechanism for the conversion of methanol to hydrocarbons followed in MFI catalysts, this suggests the rate of oxygenate conversion to olefins is slower than the oxygenate formation rate over these zeolites.^54^

At the higher Si/*T* ratio of 300, there were fewer differences in the performance of the three catalysts containing different *T* atoms in their zeolite domains. This could be attributed to the fact that the Brønsted acid site densities are too low to differentiate the performance on an acid strength basis. All of the catalysts gave approximately similar aromatic selectivities that were, on average, higher than those for the Si/*T* = 80 catalysts. The aromatic selectivity was slightly lower for the Si/Fe catalyst at lower acid site densities than higher (Si/Fe = 80, 47%; Si/Fe = 300, 37%), whereas the selectivities were higher at the low acid site density for the other two catalysts (Si/Ga = 80, 22%; Si/Ga = 300, 46%; Si/Al = 80, 13%; Si/Al = 300, 42%). The Si/Fe = 80 catalyst produced more paraffins than the other two catalysts at a similar Si/*T* ratio. The Fe-based catalysts, at both Si/*T* ratios, produced little olefins and oxygenates, showing predominantly aromatics, CO, and paraffins. The Al and Ga catalysts produced markedly higher aromatic selectivities at the low acid site density studied here (Si/*T* ∼ 300), whereas the Fe catalyst saw its performance improve at higher acid site density, where it had a measurable Brønsted acid site density. Interestingly, Al-MFI showed improved aromatics selectivity at low acid site density, where it also had predominantly or mostly Brønsted acid sites. For the Ga-based catalysts, both samples had mixed Brønsted/Lewis acidity, being ∼20% Brønsted at Si/Ga = 80 and ∼60% Brønsted at Si/Ga = 300, with the latter catalyst giving high aromatics selectivity and the former catalyst producing more paraffins. The reactivity of H-[Fe]-ZSM-5 and the production of aromatics at low acid site density with negligible Brønsted acid site density imply that the Lewis acid sites in H-[Fe]-ZSM-5 could potentially contribute on their own to the aromatization reaction or that Brønsted acidity in the mixed oxide plays a role.

### H-[B]-ZSM-5/ZnO-ZrO_2_ as a Catalyst for CO_2_ Aromatization

**Figure 6:**
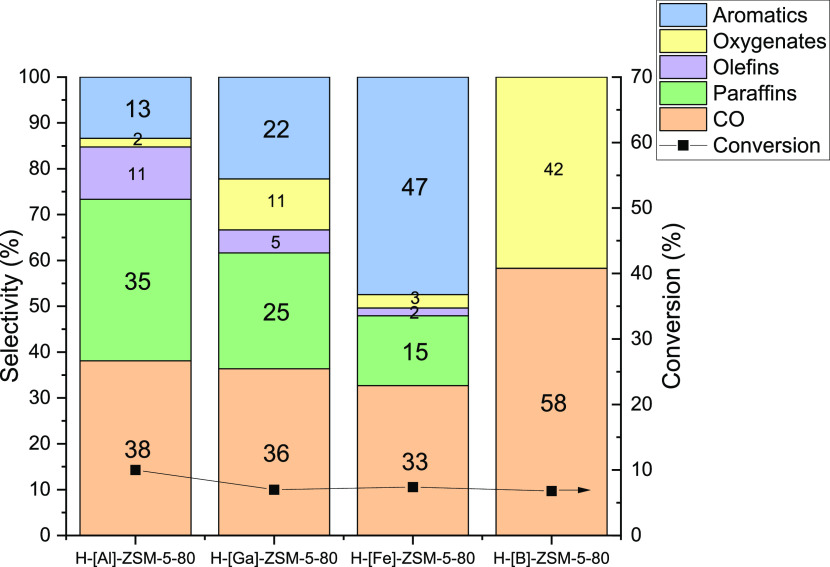
CO_2_ hydrogenation performance for H-[B]-ZSM-5–80/ZnO-ZrO_2_ with respect to H-[Al/Ga/Fe]-ZSM-5–80/ZnO-ZrO_2_. *T* = 320 °C, *P* = 600 psi, CO_2_/H_2_/N_2_ = 11:33:56, WHSV = 7200 mL g_cat_^–1^ h^–1^.

The acid site strength of H-[B]-ZSM-5 is much lower than the acid site strength of H-[Fe]-ZSM-5. In the cases of H-[Al/Ga/Fe]-ZSM-5/ZnO-ZrO_2_, it was observed that weak acid sites improve the aromatics selectivity (Figure 4). Hence, to further probe this observation, a zeolite with very weak acid sites was used to create composite catalysts. Specifically, a H-[B]-ZSM-5/ZnO-ZrO_2_ catalyst was prepared and tested for the tandem CO_2_ hydrogenation tandem reaction. As the effect of acid site strength was more apparent at Si/*T* = 80, only H-[B]-ZSM-5–80 was synthesized. Table 1 shows the zeolitic properties of H-[B]-ZSM-5–80. The acid site distributions are similar to those of the other H-ZSM-5–80 zeolites studied, and the micropore volume was similar to those of the other zeolites as well (Figure S4). The other surface characteristics were also similar to the other zeolites, confirming that apart from acid site strength, there were no significant differences among the zeolites. Figure 6 shows the CO_2_ hydrogenation results for H-[B]-ZSM-5/ZnO-ZrO_2_ with respect to the other zeolites in tandem with ZnO-ZrO_2_. It was found that there were no hydrocarbons observed in the product stream. This suggests an absence of methanol conversion, which confirms what was also observed previously in the literature.^55^ The weak acid sites of the H-[B]-ZSM-5 are so weak that they appear unable to activate methanol at the temperature of 320 °C, leading to only methanol and CO as the products of CO_2_ hydrogenation coming from the metal oxide catalyst, ZnO-ZrO_2_. Thus, from the heteroatoms tested, H-[Fe]-ZSM-5 provides the highest CO_2_ to aromatics selectivity in the case of tandem catalysts made of ZnO-ZrO_2_/H-[*T*]-ZSM-5. A weak acid site in H-ZSM-5 with enough strength to activate methanol aromatization appears to give the best selectivities for aromatics.

### Effect of WHSV on Performance of H-[Fe]-ZSM-5–80/ZnO-ZrO_2_

**Figure 7:**
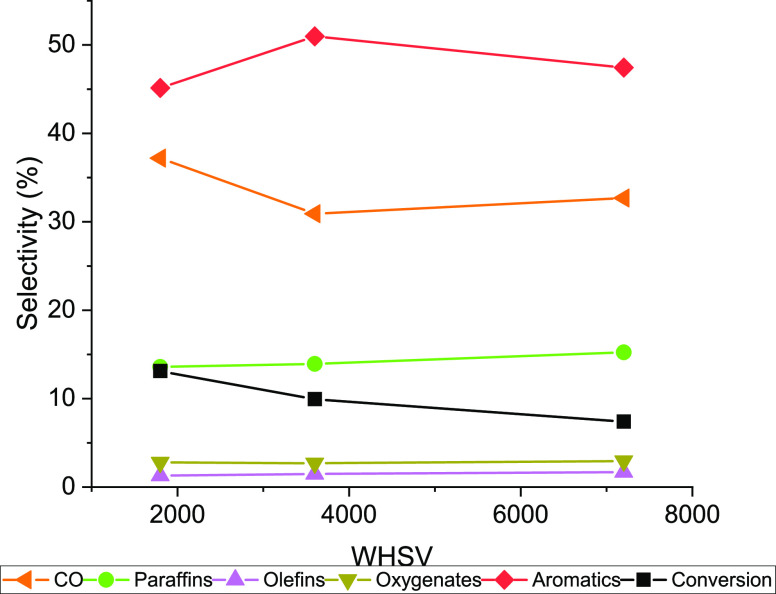
Effect of WHSV on performance of H-[Fe]-ZSM-5–80/ZnO-ZrO_2_ for CO_2_ conversion. *T* = 320 °C, *P* = 600 psi, CO_2_/H_2_/N_2_ = 11:33:56, WHSV = 7200 mL g_cat_^–1^ h^–1^.

H-[Fe]-ZSM-5–80/ZnO-ZrO_2_ showed the highest CO_2_ to aromatic selectivity among the catalysts tested. As the reaction was initially tested in a low-conversion, differential reactor, the effect of WHSV was evaluated over this catalyst at higher conversions. Figure 7 shows the catalytic performance with a changing WHSV. With a decreasing WHSV, the CO_2_ conversion increased significantly and almost linearly. The aromatics selectivity across the three WHSVs tested remains similar, with any variations in selectivity seeming to be balanced by concomitant variation in CO selectivity. Low oxygenate selectivity shows that any methanol synthesized by ZnO-ZrO_2_ is largely converted to hydrocarbons over these molecular sieve domains. This may imply that methanol synthesis is the rate-determining process under these CO_2_ conversion conditions. The increase in CO selectivity is consistent with the increase in CO selectivity for CO_2_ conversion over ZnO-ZrO_2_ alone, as observed in our earlier study (see Figure S11).^48^ Of the hydrocarbons produced, paraffin selectivity increased at the low WHSV of 1800.

### Catalytic Stability of H-[Fe]-ZSM-5–80/ZnO-ZrO_2_

**Figure 8:**
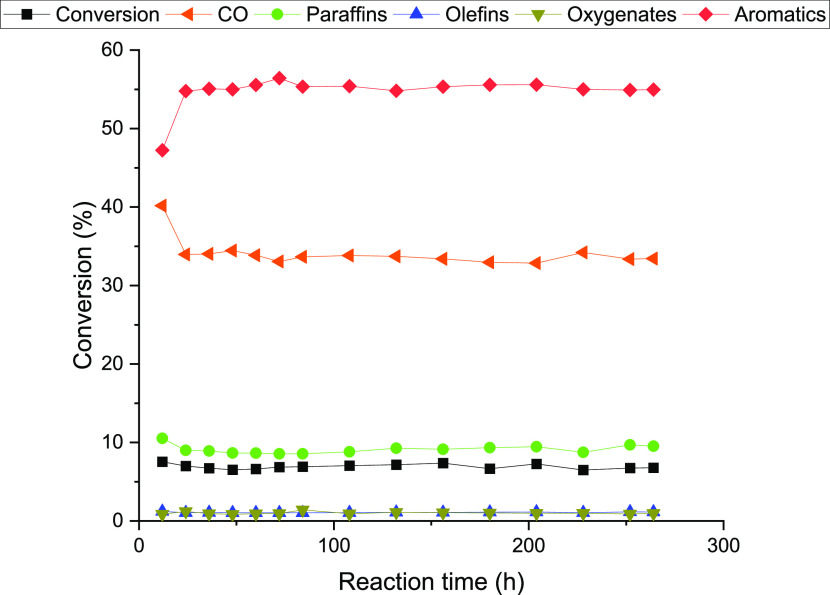
Catalytic stability of H-[Fe]-ZSM-5–80/ZnO-ZrO_2_ under CO_2_ hydrogenation. *T* = 320 °C, *P* = 600 psi, CO_2_/H_2_/N_2_ = 11:33:56, WHSV = 7200 mL g_cat_^–1^ h^–1^.

The H-[Fe]-ZSM-5–80/ZnO-ZrO_2_ catalyst was tested for its stability at extended time on stream–up to 264 h (Figure 8). The catalyst performance stabilizes around 24 h, after which the catalyst shows very similar performance over the complete period of testing. This testing shows that the catalyst does not deactivate over more than 10 days of operation and continues to give a high CO_2_ to aromatics selectivity throughout the duration of the test. Tandem catalysts based on H-ZSM-5/ZnO-ZrO_2_ have previously been shown to be stable under CO_2_ hydrogenation^30,31,33^ conditions and the change of *T* atoms from Al to Fe seems to have no detrimental effect on the catalytic stability over the period studied.

## Conclusions

These collected observations led to several concluding points. ZnO-ZrO_2_/H-[Fe]-ZSM-5–80, with CO_2_ to aromatics selectivity of ∼51% (Figure 7), is the highest observed so far in the literature for similar reaction conditions and CO_2_ conversions in the methanol-mediated conversion of CO_2_ to aromatics.^13^ It is also evident based on the results with this composite catalyst that H-ZSM-5 with weak acid sites can give good aromatics selectivity under the conditions employed. However, we cannot conclude that predominantly Brønsted acid sites are necessary for the reaction, even though a minimal migration of Zn^2+^ ions was observed (Figure 5). This is because the Lewis acid sites of H-[Fe]-ZSM-5 alone appear to offer sites capable of the aromatization reaction, as seen in the literature in the case of some Ga-MFI samples, as well.^45^ For catalysts with seemingly few inherent Brønsted acid sites, it is also possible that Brønsted sites are produced in situ, under reaction conditions, by water (produced in the RWGS reaction) sorption on Lewis sites. For catalysts with stronger Brønsted acid sites, such as those based on Al substitution, our results are consistent with literature trends, as well as our prior work. High acid site densities lead to lower aromatics selectivities, whereas intermediate values of Si/Al (Si/Al = 200–400) improved the performance.^48^ As before, the Brønsted acid-rich Si/Al = 300 catalyst gave excellent aromatics selectivity. For the Ga-based systems, which are known to nearly always give mixtures of Brønsted and Lewis sites, aromatics selectivity was markedly improved at lower acid site density (46 vs 22%). Overall, high aromatics selectivity is best correlated to low total acidity, a balanced Lewis/Brønsted acid site ratio, and weak acid sites. Zeolites with extremely weak acid sites, H-[B]-ZSM-5, are not able to activate methanol to make hydrocarbons, leading to the H-[Fe]-ZSM-5 being the best candidate of the zeolite catalysts tested for the tandem CO_2_ to aromatics conversion reaction with ZnO-ZrO_2_. Using the best catalyst ZnO-ZrO_2_/H-[Fe]-ZSM-5–80, CO_2_ conversions up to 13%, with minimal effect on the aromatics selectivity (∼45 vs 51% obtained at lower conversions), were obtained by changing the WHSV of the feed.

## Supporting Information Available

Schematic for process setup; X-ray diffraction studies; SEM; particle size distribution; N_2_ physisorption; ^27^Al MAS NMR; ^71^Ga MAS NMR, diffuse reflectance UV/vis spectrometer studies, ^29^Si ssNMR, H_2_ TPR, and EDS analysis; and catalytic testing (PDF)
