# Supplementary material for: Isomorphous Substitution in ZSM-5 in Tandem Methanol/Zeolite Catalysts for the Hydrogenation of CO2 to Aromatics
Source: Energy Fuels. 2024 Jan 9;38(3):2224–34. doi: 10.1021/acs.energyfuels.3c03755 (PMC10839831; doi:10.1021/acs.energyfuels.3c03755)
Supplement: Supplementary file 1 — ef3c03755_si_001.pdf [file ef3c03755_si_001.pdf]

Supporting Information for

Isomorphous Substitution in ZSM-5 in Tandem Methanol/Zeolite Catalysts for Hydrogenation of CO_2 to Aromatics

Dhrumil Shah,[a,#] Iman Nezam,[a,#] Wei Zhou,[a,b], Laura Proaño,[a] Christopher W. Jones[a*]

[a] School of Chemical & Biomolecular Engineering, Georgia Institute of Technology, 311 Ferst Dr., Atlanta, GA 30332, United States

[b] State Key Laboratory of Physical Chemistry of Solid Surfaces, Collaborative Innovation Center of Chemistry for Energy Materials, National Engineering Laboratory for Green Chemical Productions of Alcohols, Ethers and Esters, College of Chemistry and Chemical Engineering, Xiamen University, Xiamen 361005, P. R. China.

[#]these authors contributed equally to this work

*cjones@chbe.gatech.edu

Catalytic Reactor

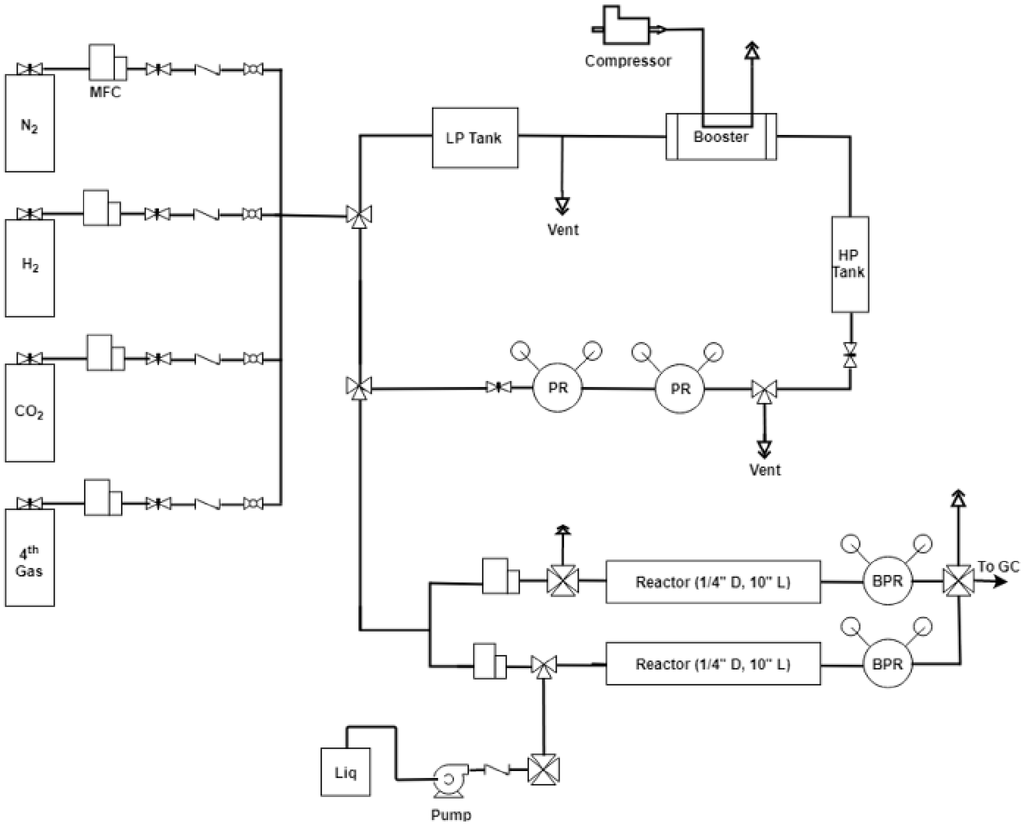

Figure S1: Reactor configuration for catalytic testing for CO_2 hydrogenation.

Catalyst Characterization

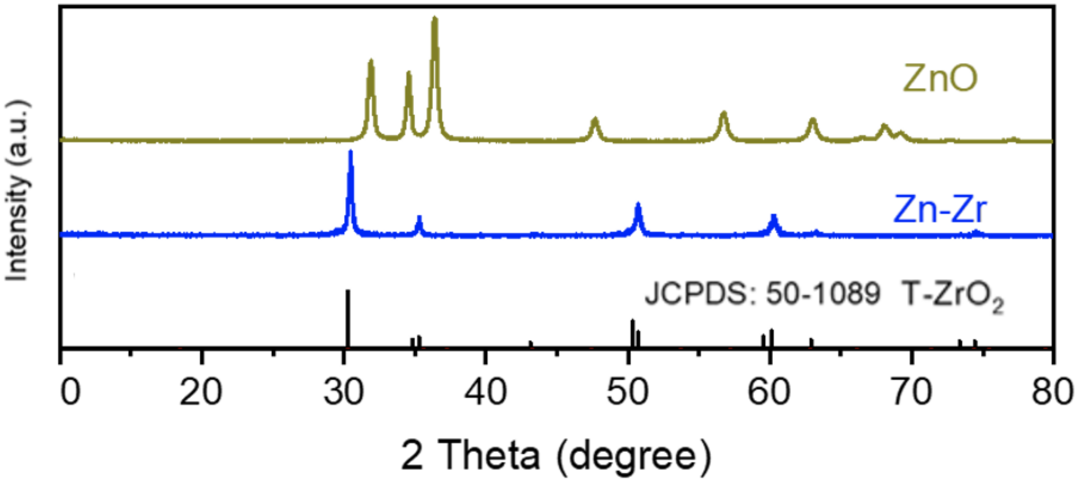

Figure S2: XRD patterns of ZnO-ZrO_2 (1/6 Zn/Zr molar ratio), commercial ZnO, and tetragonal ZrO_2.

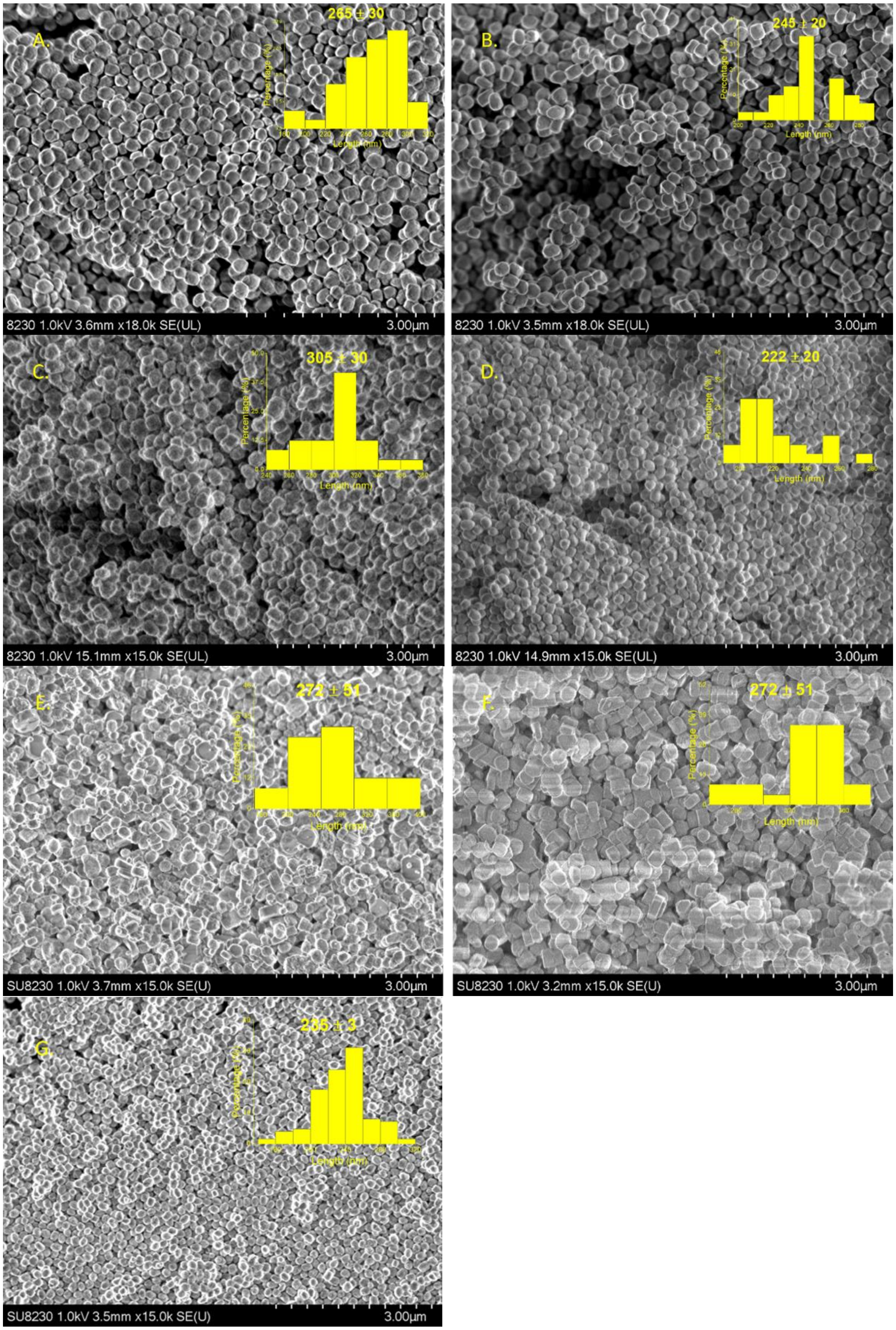

Figure S3: Scanning electron microscopy (SEM) images of H-ZSM-5 samples (A) H-[Ga]-ZSM-5-80, (B) H-[Ga]-ZSM-5-300, (C) H-[Fe]-ZSM-5-80, (D) H-[Fe]-ZSM-5-300, (E) H-[Al]-ZSM-5-80, (F) H-[Al]-ZSM-5-300 (G) H-[B]-ZSM-5-80.

Table S1: Particle size distribution of synthesized H-ZSM-5 samples

H-ZSM-5	Particle size (nm)	Standard deviation (nm)
H-[Fe]-ZSM-5-80	305	30
H-[Fe]-ZSM-5-300	222	20
H-[Ga]-ZSM-5-80	265	30
H-[Ga]-ZSM-5-300	248	20
H-[Al]-ZSM-5-80	272	51
H-[Al]-ZSM-5-300	329	29

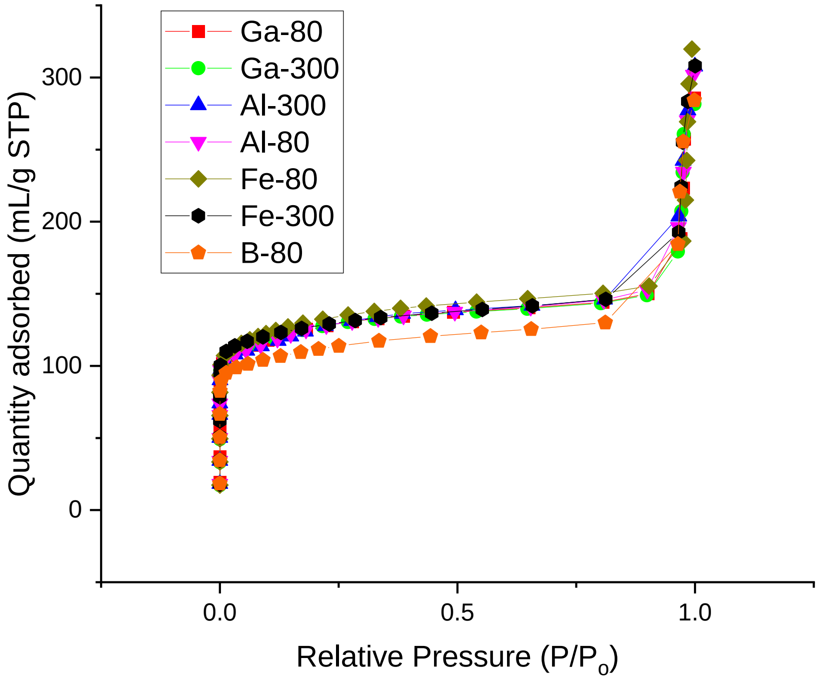

Figure S4: Nitrogen physisorption isotherms of H-ZSM-5.

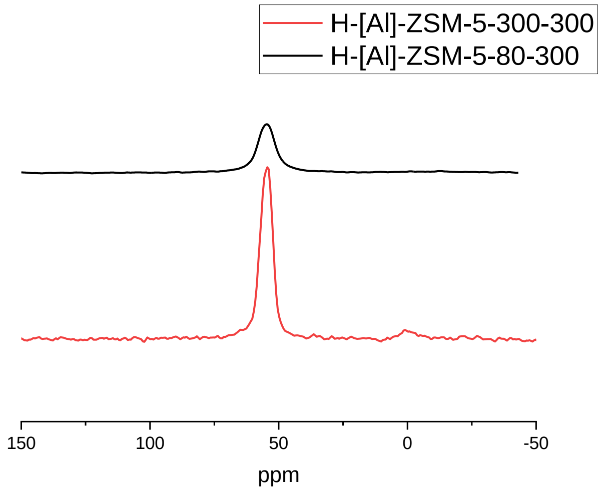

Figure S5: ^{27}Al ssNMR for H-[Al]-ZSM-5.

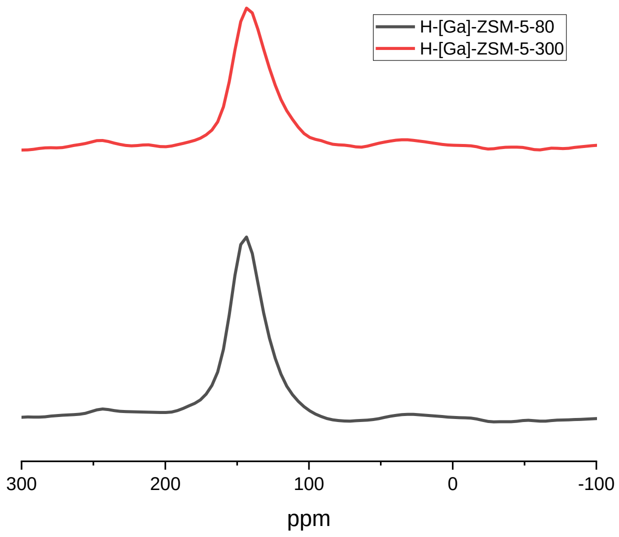

Figure S6: ^{71}Ga ssNMR for synthesized H-[Ga]-ZSM-5

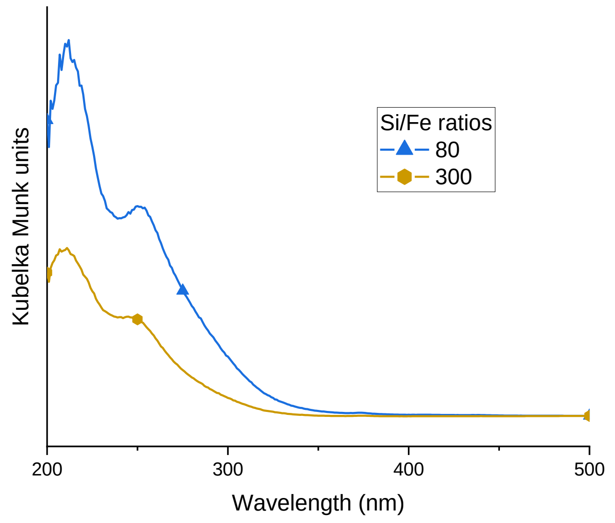

Figure S7: Diffuse reflectance UV/Vis spectrometer studies for synthesized H-[Fe]-ZSM-5.

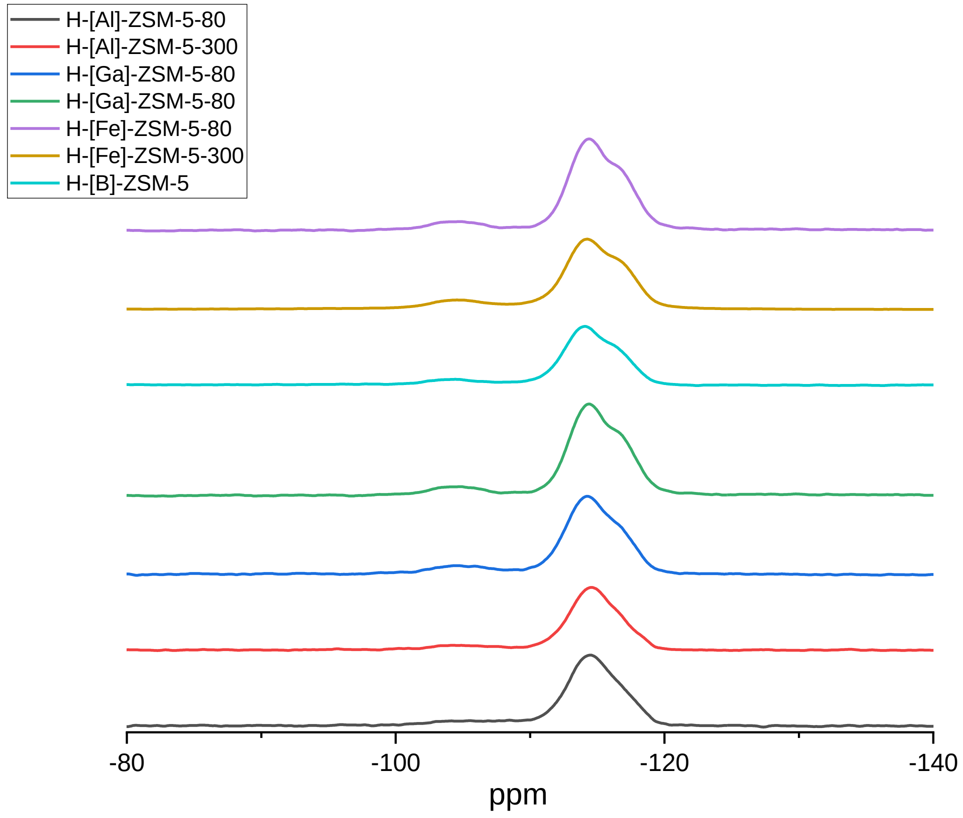

Figure S8: ^{29}Si ssNMR for H-[T]-ZSM-5.

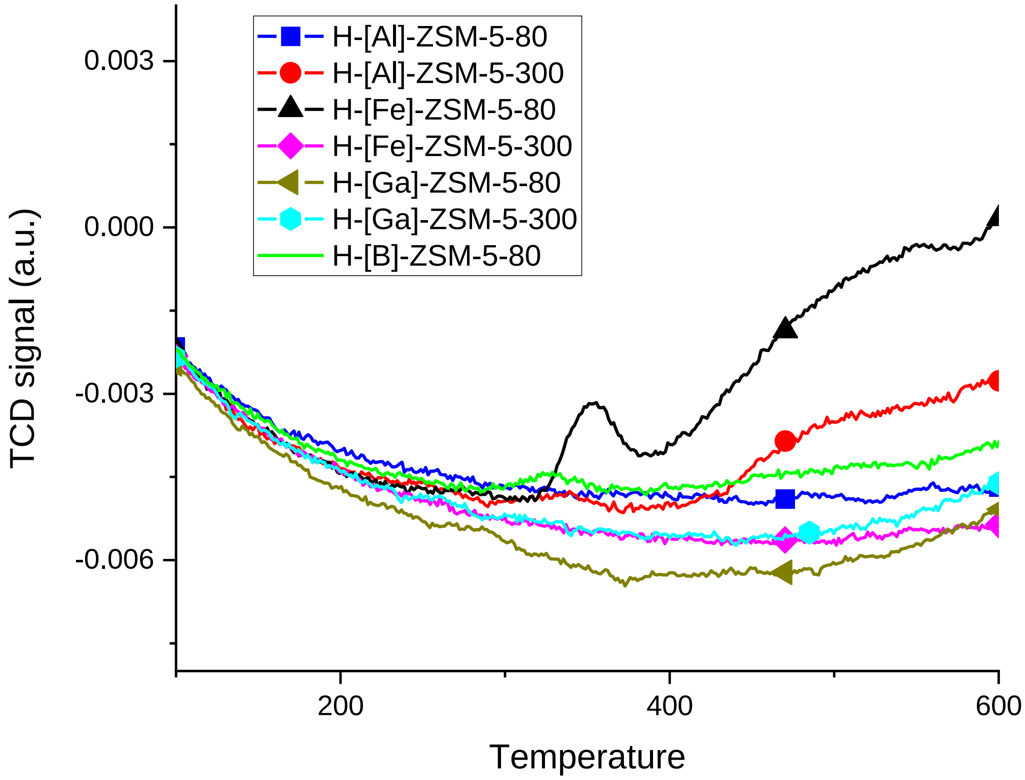

Figure S9: H_2-TPR data of synthesized H-[T]-ZSM-5s

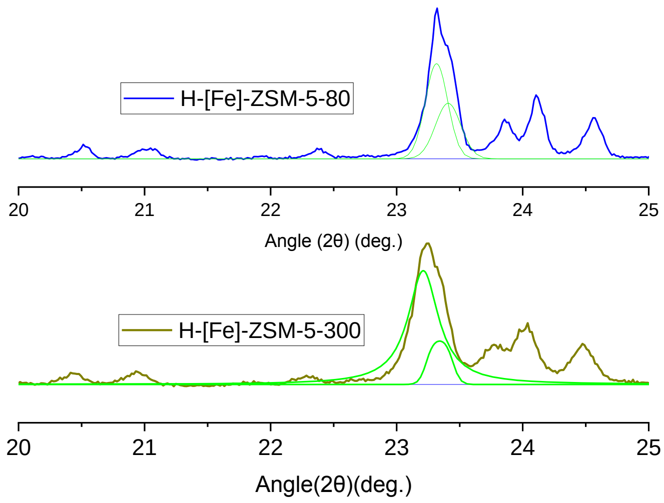

Figure S10: Deconvolution of X-ray diffraction peaks of H-[Fe]-ZSM-5

Catalytic Results

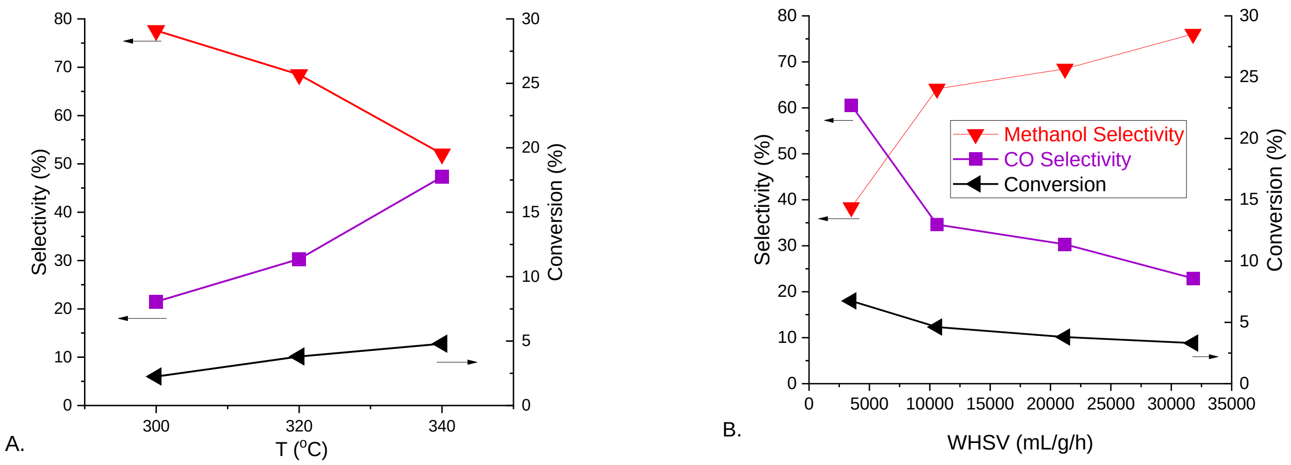

Figure S11: Catalytic performance of the ZnO-ZrO$_2$ catalyst as a function of (A) temperature at WHSV = 21600 mL $g_{cat}^{-1}h^{-1}$, and (B) WHSV at 320 °C. P = 600 psi.

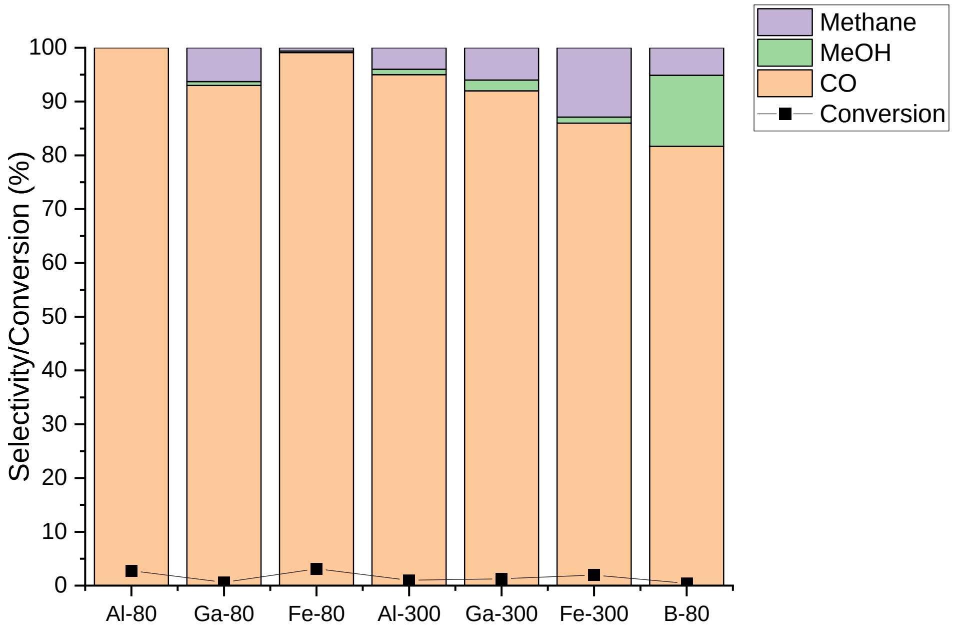

Figure S12: CO$_2$ hydrogenation over H-[T]-ZSM-5-x in the absence of ZnO-ZrO$_2$ (x-axis labelled in the format: "T"-"x")

Table S2: Experimental standard deviation of estimated products selectivities (%) for CO_2 hydrogenation reaction on H-[T]-ZSM-5/ZnO-ZrO_2.

**H-[T]-ZSM-5/ZnO-ZrO_2**	**Error (CO) (%)**	**Error(Paraffins) (%)**	**Error(Olefins) (%)**	**Error(Oxygenates) (%)**	**Error(Aromatics) (%)**
H-[Al]-ZSM-5-80	1.41	1.10	0.42	0.58	1.27
H-[Al]-ZSM-5-300	2.57	4.20	4.28	0.86	1.63
H-[Ga]-ZSM-5-80	4.03	4.25	1.53	0.11	1.42
H-[Ga]-ZSM-5-300	3.28	2.22	0.31	1.36	0.59
H-[Fe]-ZSM-5-80	0.76	0.82	0.18	0.90	0.86
H-[Fe]-ZSM-5-300	1.07	3.72	0.85	2.77	2.87

Experimental error on product selectivities is calculated using the following formula:

$$Error_i = \sqrt{\frac{\sum_{i=1}^{n}(y_{i,avg} - y_{i,j})^2}{n}}$$

Where, $y_{i,j}$ represents % selectivity of product i, for j^{th} repeated experiments for same conditions and catalyst, while $y_{i,avg}$ is the average % selectivity of the product i over n experiments done for a particular catalyst and reaction condition.
